# How Outstanding Universal Value Attractiveness and Tourism Crowding Affect Visitors’ Satisfaction?

**DOI:** 10.3390/bs13020112

**Published:** 2023-01-29

**Authors:** Sifeng Nian, Min Chen, Xiaowan Zhang, Donghe Li, Jingya Ren

**Affiliations:** 1 School of Business, Anhui University, Hefei 230601, China; 2 Center for Hui Studies, Anhui University, Hefei 230039, China; 3 School of Geographic and Oceanographic Sciences, Nanjing University, Nanjing 210023, China

**Keywords:** tourist satisfaction, outstanding universal value, tourism crowding, destination attachment, World Heritage Site

## Abstract

A World Heritage Site is a masterpiece of mankind and/or nature that possesses outstanding universal value (OUV). In this regard, the 5Cs strategic objectives (credibility, communication, capacity-building, conservation, and community) set by the World Heritage Committee have become a main issue for WHS sustainable development. As one of the key stakeholders of WHS, tourist’s perceived OUV attractiveness, congestion, and attitudinal behavior have significant implications for heritage protection and tourism’s sustainable development. Based on the perspectives of OUV attractiveness and perceived tourist crowding, and taking into account destination attachment, the influencing factors and mechanisms of tourist satisfaction are investigated. In view of the 536 questionnaire responses from tourists of Mount Sanqingshan National Park, the structural equation modeling approach was employed to study tourist satisfaction. The conclusions were sketched: (1) tourist crowding perception did not have a significant negative effect on OUV attractiveness; destination attachment, and tourist satisfaction, and the degree of crowding perception was low; (2) the OUV attractiveness has a significant positive influence on destination attachment and tourist satisfaction, which fully highlights the charm of OUV and its important role in shaping tourists’ attitudes/behaviors; (3) destination attachment has a significant positive effect on tourist satisfaction, indicating that tourists’ heritage-place attachment contributes to tourist satisfaction. Finally, the analysis of tourism crowding, OUV, and the satisfaction framework proposed broaden the horizons of visitor satisfaction research, which is also a positive response to the strategic objectives of the 5Cs of WHS, with some practical implications for heritage preservation and visitor management in World Heritage Sites.

## 1. Introduction

World Heritage Sites are the best cultural treasures and natural wonders of all mankind, bridging the gaps of dialogue and exchange among civilizations. World Heritage Sites (WHS) are rare and irreplaceable treasures of mankind designated by UNESCO and the World Heritage Committee; each is a cultural heritage and/or natural landscape acknowledged by all mankind as having outstanding meaning and universal value [1]. This is also the basic requirement of the convention concerning the conservation of the world cultural and natural heritage, and outstanding universal value (OUV) is the core criterion and the most significant basis for nominations to become WHS [1]. The status of the World Heritage Site is not only a place of the highest value in need of protection, it is also a hot spot of iconic value to the tourist industry and for regional economic growth [2]. The WHS is a world-class “famous brand”; once a place is successfully nominated, the “golden flagship” will become a popular tourist destination both nationally and globally, and the nomination will, in particular, usher in the rapid development of tourism. By July 2021, the overall amount of WHS had reached 1122, dispersed throughout 167 nations. China has 56 WHS; along with Italy, it has the most WHS projects. The great benefits brought by the “money tree” of WHS have been accompanied by tremendous pressure and even threats triggered by the rapidly expanding tourist industry, which poses a particularly serious problem for tourist development at the WHS. Because a nominated WHS is a nationwide “tourist highlight” or “must-visit” attraction, it is deemed a tourist magnet [3]. Just as Caust et al. wonder, is it a blessing or a burden to be listed as a WHS in developing Asian countries [4]? It can be said that the World Heritage Site has a high reputation and at the same time is under great pressure for tourism development.

In particular, China’s huge population (1.41 billion people in mainland China in 2021) and its rapid economic development since its reform and opening up (from 1978 to 2018, the average annual growth rate of GDP was 9.5%) have generated considerable demand for leisure tourism (the number of domestic tourists reached 6.0 billion in 2019), and a rapid increase in the volume of international tourists in recent years. As an outcome, the number of tourists visiting WHS has skyrocketed. For example, Mount Sanqingshan National Park has seen its tourist numbers soaring from 1.48 million in 2008 to 23.95 million in 2019, following its WHS listing in 2008. Generally, the World Natural Heritage Sites are ecologically fragile and vulnerable to ecological pressure and threats, which often face issues such as tourism crowding, capacity overloading, overdevelopment of tourist facilities, serious over-commercialization and adverse impacts, all of which pose major challenges to OUV conversation and the sustainable development of WHS [5,6,7]. It is especially important to measure the impact of crowding and protect OUV in the face of excessive visitors, resulting in congestion and capacity overload [8,9,10]. Academics have researched the association to WHS conservation and tourism development primarily from the perspective of government behavior, organization strategy, community involvement, and the performance of other stakeholders [11,12]. Since the large number of tourists and their attitudes and behaviors will have a certain influence on the conservation and sustainable development of WHS, tourists, as important stakeholders in WHS protection and tourist crowding, have a direct emotional response to, and influence on, the ecological, psychological, infrastructural, and social capacity. Tourists are the agents of ecological, environmental, low-carbon, and sustainable tourism, as well as being negative influences of various forms. Tourists are not only the beneficiaries and experiencers of heritage protection; they are also participants, executors, and contributors to preservation, as well as supervisors and eyewitnesses. How do tourists feel about an OUV attraction when they experience the heritage value? How does tourist crowding relate to their satisfaction, and affect their OUV perception, destination attachment, and heritage conservation? What factors motivate their inner state and emotional changes as they experience the development of heritage tourism? These are the starting points and emphasis of this study.

WHS visitors are naturally linked to WHS conservation and sustainable development; scholars have studied tourism conflict incidents, the complex relationship that exists between tourism and World Natural Heritage Sites, and the motivation, quality of experience, and satisfaction of tourists on WHS trips [13]. Much attention was paid to tourist attitudes and behaviors in terms of tourist crowding, capacity congestion, OUV attractiveness, place attachment, and satisfaction [9,10,14,15,16,17]. From the point of view of the attractiveness of the OUV, its value has been highlighted [18,19]. From an active brand marketing perspective, the WHS title could entice tourists and generate shared awareness, and the OUV attraction plays a key role in tourist appreciation and loyalty to the tourist destination [13,19]. The all-encompassing appeal of the WHS had a considerably positive effect on tourist place and destination attachment, which helps to promote heritage protection and tourist satisfaction [20,21,22,23,24]. Researchers have examined the influence of the connections between tourists’ place, heritage, and destination attachment on heritage preservation and tourism satisfaction [20,25,26,27,28]. Furthermore, studies have been conducted from the perspectives of tourists’ perceived value, experience and service quality, satisfaction, environmentally responsible behavior, and WHS protection [20,27,29,30,31,32]. Tourism is an experiential activity and an emotional process; tourists’ satisfaction and loyalty have a positive emotional influence on heritage protection [33]. Related research topics associated with it have received academic attention, such as place attachment [22,34], perceived value [29,35], service quality [36], satisfaction [37], value experience perception [29,35], and environmental behavior [26]. Meanwhile, related scholars studying how congestion and crowding affect destination attractiveness, place attachment, and satisfaction have come up with mixed results; factors such as crowding and overtourism have a negative impact on the attractiveness of tourist destinations [15,17,38], reducing the effect of positive emotions on visitors [6]. This directly affects the quality of tourists’ travel experience, destination attachment and satisfaction, and physical crowding and psychological crowding in different tourist destinations show different representational relationships [7,9,39,40,41]. Research has also been conducted on the attitude and intention of heritage tourism, place attachment [22], perceived tourism value [29,35], and visitor experience and resource protection [42], in order to better interpret the influence of tourists in WHS. As to the factors influencing tourists’ overall satisfaction and their pathways, these have important implications for heritage conservation, destination tourism capacity, visitor management, and sustainable development [22,37,43]. Visitors’ attitudes and behaviors can be seen to be influenced by a variety of factors, and these have important implications for the study of visitor satisfaction at World Heritage Sites.

We constructed the conceptual framework based on tourist satisfaction to a heritage tourist destination—utilizing structural equation modeling as our methodology and Mount Sanqingshan National Park as our case study area—to explore the correlations between tourists’ attitudes and behaviors towards tourism crowding, OUV attractiveness, destination attachment, and to further explain the affecting determinants and mechanism of visitors satisfaction in WHS, with a view towards benefitting heritage protection and tourist sustainable development. In the following sections, we provide an overview of the crowding, OUV, and satisfaction framework, review the concepts of relevant core dimensions, and propose research hypotheses. The next section introduces the research method and data analysis. Finally, we discuss the results and potential implications and provide study limitations and future study prospects.

## 2. Literature Review and Conceptual Framework

### 2.1. Tourist Crowding Perception

Crowding is an important area of study in environmental psychology, which connotes the subjective experience of density and spatial constraints [44]. Crowding influences mainly include physical, social, and personal factors, and crowding can lead to excessive physiological arousal, negative social behaviors, and dysmotivation [45,46]. Existing crowding theories have interpreted crowding from several perspectives, including environmental stimuli and individual behavioral responses. The study of crowding in tourism contexts began to receive extensive attention with the great development of outdoor recreation in the 1850s, focusing mainly on psychological and physical crowding [45,46]. The phenomenon of overcrowding has become a dilemma for scenic spot development, destroying the quality of the scenic environment and reducing the quality of tourists’ leisure experience; in particular, tourist overcrowding causes significant pressure on the OUV of WHS, and even undermines conservation of heritage and sustainable development to WHS sites [47]. 

Tourist crowding is essentially the excessive concentration of tourist flows in a specific spatial and temporal unit, which impacts the quality of the tourist experience [48]. The seasonal differences and spatial non-uniformity of tourist activities result in tourist congestion, which usually occurs during peak tourist seasons such as public holidays such as golden week, and is spatially concentrated in a few popular tourist attractions or travel destinations such as WHS, National Parks, and World Geoparks [9,15]. There are significant differences in visitor crowding, spatial scenes and crowd environment, etc., which are likely to have a negative influence on visitor emotion, tourism satisfaction, and loyalty, and to trigger product switching, rationalization, replacement, and other adjustment behaviors [15,49,50]. Related scholars have advanced from various perspectives, including the connotation, measurement, generation mechanism, influencing factors, and effect of tourist crowding, involving expectancy theory, social interference theory, social identity theory, stimulus overload theory, and density reinforcement theory [47,51].

Regarding the influential aspects of tourism congestion, the relevant influencing factors are related to contextual factors, personal factors, external factors, and other factors [10,17,52]. Visitor crowding is a multidimensional concept, where crowding perception is considered as a comprehensive evaluation of the surrounding environment from both physical and psychological viewpoints, and Stokols proposed two dimensions of physical crowding and social crowding [53]. Among them, physical crowding refers to the sense of space shortage due to non-personal factors, and social crowding refers to the sense of lack of space due to the number of individuals and social interactions between individuals in a certain environmental context. Gramann et al. categorized the crowding experience of outside recreation into physical crowding, behavioral crowding, and goal-related crowding [54]. Desor pointed out that the broad psychological correlates between the population size and the general degree of social stimulation, which are assumed to be the variables controlling human judgments of crowding, turn out to indicate that crowding criteria change with ongoing activity [44]. Crowding is an evaluative attitudinal concept; intensive crowding probably accompanies negative assessments, and stressful mental stimuli can influence crowding judgments and may happen while personal verges are crossed [41,46,55]. Increased crowding can lead to lower expectations and satisfaction, resulting in increased avoidance behaviors for unfavorable crowding assessments, which may reduce enjoyment of the destination experience [55,56]. It can be said that tourist crowding has a major impact on tourist satisfaction—there are generally negative, positive, and no correlations—such as the negative effect of overcrowding and overtourism on visitor satisfaction and loyalty [10,38,52]. As the feeling of crowding increases, it can reduce visitor satisfaction [57] or have no significant effect on crowding perception on satisfaction [58]. Several scholars have examined the mediating or moderating mechanisms, such as accommodation strategies in the relationship between the perception of crowding and satisfaction [56], a significant negative influence of crowding perception on visitors’ evaluation of a scenic spot, and a significant positive moderating effect of age [15]. Tourist satisfaction is a comprehensive evaluation of tourist products, tourist services, tourist destinations, etc. The link between tourist crowds and satisfaction has been debated, which show a differentiation. Visitor crowding is significantly negatively related to satisfaction [57]; crowding is found to decrease visitor satisfaction when perceived by visitors [59]; visitor crowding perception is not related to satisfaction, and perceived crowding results in coping behavior, which in turn rises crowding in the influenced population perception [56].

Meanwhile, the analysis was conducted from the perspective of tourist crowding and tourists’ emotions. Crowding, as the most intuitive feeling of tourists at the destination or during the tour, is closely associated with changes in tourists’ affective attitudes. Shi et al. assessed destination attractiveness by the number of photos posted by tourists on social media platforms to investigate the impact of crowding on tourists’ emotions, and found that crowding led to positive emotions [50]. Sharp et al. investigated tourists’ perception of crowding and place attachment and found no remarkable association amid tourist crowding and destination attachment [39]. Liu et al. used five positive words (happy, good, relaxed, cheerful, content) and three negative words (mad, angry, irritated) in combination with interviews to explore the positive and negative emotions caused by crowding and found that crowding was not always a “terrible” outcome [49]. Visitor crowding is strongly associated with visitor loyalty, and crowding is an important influencing factor in encouraging or dissuading visitors to revisit or recommend [41,55]; it is generally considered to have a significant negative effect on visitor loyalty [60]. Studies have shown that tourist crowding and overtourism have a significant negative effect on destination attractiveness and attachment [15,17]. In addition, it is hypothesized that the effect of crowding on destination attractiveness would likely impact visitors’ emotional response [38]. Tourist crowding decreases the positive impact of previous visitors on present arrivals, signifying that increased crowding may undermine destination attractiveness [6]. Crowding reduces recreationists’ destination attachment and has a negative effect on the quality of tourist feeling, changing with different stages of destination life cycle, especially in popular and famous World Heritage destinations [7,9,40,41].

In summary, tourism crowding perceptions play a crucial role in tourist satisfaction, destination attractiveness, and place attachment, while presenting different attitudes at various stages of destination life cycle and in different crowding contexts. We propose the resulting hypotheses:

**Hypotheses** **1** **(H1).** *Tourist crowding has a negative impact on tourist satisfaction.*

**Hypotheses** **2** **(H2).** *Tourist crowding has a negative impact on OUV attractiveness.*

**Hypotheses** **3** **(H3).** *Tourist crowding on has a negative impact on destination attachment.*

### 2.2. Outstanding Universal Value Attractiveness

The OUV is related to cultural and/or natural importance so extraordinary that it exceeds national limits and is of common significance to present and upcoming generations of all humankind [1]. The nomination as a WHS aims to protect people’s common property, which is of great significance to the entire international community. The OUV is the chief foundation for its nomination, including 10 assessment standards, with integrity and/or authenticity, and sufficient preservation and management mechanisms that ensure the WHS site is sheltered and could be deemed the OUV [1]. The World Natural Heritage Sites refers to a natural resort or an obviously described natural zone with OUV from the view of science, conservation, and natural advantage, which is the maximum straight appearance of WHS attraction and essential attractiveness for tourist growth, and which is also the key effort of WHS preservation [2,61]. The attractiveness of a travel resort is a key feature of tourism academic research, whether from perceived emotional experience or a functional service perspective, and OUV is certainly central to the site [62]. Destination attractiveness refers to how a destination meets a person’s needs or personal perceived benefits, and consists of core and extended attributes [63] which have a great attraction to tourists [12]. Meanwhile, tourist congestion and crowding reduce the positive impact of previous visitors on present arrivals, proposing that increased crowding may worsen the attractiveness of the resort [6]. Visitor loyalty is achieved through destination personality, satisfaction, and identity, and the results show that destination personality promotes visitor satisfaction, visitor identification with the destination, positive word-of-mouth, and willingness to revisit. The core qualities relate to distinctive natural and humanistic capitals, just as OUV, while extended qualities refer to functional features such as well-functioning tourist amenities, care services, and highly effective organizational management in WHS. In general, the two complement each other and jointly enhance a heritage site’s attraction to tourists. Employing USA WHS as case research, Hazen examined the perception measurement of tourists’ OUV, showing that the aesthetic, cultural, educational, environmental, reactional, and spiritual value aspects of a WHS could aid improve its heritage conservation [19]. Baral et al. adapted Mt. Everest National Park to measure the OUV and visitors’ perceptions of the OUV, containing importance, individuality, influence, legacy, values, and appeal [64].

Related scholars have studied the combined effects of destination distinctiveness, tourism attraction systems, and destination attractiveness on tourists, and the results show that destination attractiveness plays an important role for tourists, especially in terms of their satisfaction and willingness to revisit, as well as through value co-creation with WHS sites in order to better attract potential tourists [65,66]. Tourist attractiveness is considered the core of the tourism process, i.e., the main reason to visit a specific destination, providing a series of activities and good experiences for tourists and becoming an important means of collecting signs of consumption. It can be said that the impulse to travel in order to witness ‘extraordinary’ or ‘marvelous’ objects seems to be deep in all human cultures. Studies have shown that WHS status, the wealth of the place, its ecology, and its openness to external markets, influences the attractiveness of a destination to tourists and is also a mutual achievement that goes beyond the mere preservation of the heritage itself [67]. For WHS sites, their OUV has a great impact on tourists; however, at the same time, there is also the phenomenon of losing the perceived value of tourists, such as via insufficient protection of OUV, poor interpretation, and the existence of cross-cultural communication difficulties and the conflict between global and local contexts. Theme, product, and design are important attractions of heritage tourism, which influence the decision-making processes of visitors, while interactive elements are the biggest influences on self-learning and entertainment; participation experience affects visitors’ perception and satisfaction; in particular, the authenticity of heritage sites has a substantial effect on visitor satisfaction and loyalty, as well as attractiveness and heritage value. The quality of performance of a tourist destination has an important effect on satisfaction, benefits, and behavioral intentions. Tourism crowding is an important factor in the perceived impact of resort and OUV attractiveness, and can also reduce tourist satisfaction and diminish visitors’ willingness to pay [55,56,57,59].

Investigation has shown that OUV attractiveness has a key impact on tourist loyalty to the site, experience of value perception, environmentally conscious behaviors, and attitudes and behaviors towards heritage protection [14,22,28,68]. Even the attraction of ordinary tourist destinations had a key positive effect on visitors’ destination attachment [21]. A destination’s main attraction, and its secondary attractiveness, such as tourism services and native communities, had positive influences on tourist resort attachment, experience of perceived tourist values, and tourists’ attitudes and behaviors with respect to defending the tourist destination [69]. Chi et al. checked the structural relationship between destination image, visitor satisfaction, and resort loyalty, where destination image, including tourism environment, natural attractiveness, recreation and activities, and historical attractiveness, has a profound impact on visitor satisfaction and loyalty overall [70]. Research by Reitsamer et al. demonstrated that embodied perceptions of tourist resort attractiveness could improve destination attachment and sensed tourism values, changing visitor’s attitudes, and thus improved visitors’ satisfaction [71]. The attractiveness of WHS has a positive impact on visitor motivation, promoting tourist feeling and rising pertinent wisdom, which could stimulate and transform the inner condition of visitors [72]. Destination attractiveness could intensify tourist destination attachment and indorse environmentally responsible behavior, enlarging tourist effectiveness and visitor satisfaction [73]. As a core charm of WHS, the OUV has a valuable impact on how visitors experience value, tourism motivation, tourist satisfaction, and connectedness with the destination. Perceived OUV attractiveness had a positive influence on destination attachment, which revealed that the essential OUV attraction of the World Natural Heritage Sites had a valuable effect on visitors’ experience of worth and emotional affection [13,19,22,74]. Tourist destination attractiveness affects their travel intentions, pleasure, and place attachment, while tourist satisfaction, interactions, and revisits also enhance perceived tourist attractiveness of the destination [75]. Destination attractiveness and destination attachment are also influenced by accessibility, local community, and scenic beauty, while destination source credibility plays a key position in destination attachment and destination satisfaction [71]. Consequently, the hypotheses were raised:

**Hypotheses** **4** **(H4).** *OUV attractiveness has a positive impact on destination attachment.*

**Hypotheses** **5** **(H5).** *OUV attractiveness has a positive impact on tourist satisfaction.*

### 2.3. Destination Attachment

Tuan points out that sense of place is the characteristic of “site” itself and human’s affection to location, noting that the subsequent development of experience, memory, and intention leads to the formation of a deep attachment to place, which is referred to as place attachment [76]. Williams proposed that place attachment consists of place identity and dependence, where the former is a functional attachment amid human and earth, and the latter is an emotional attachment [77]. The notion of place was together physical and psychological, and was explained, sensed, comprehended, and envisaged in the narrative [78]. Tourism and travel are key ways for people to sense and comprehend the setting as an intersection of themselves, and the place with significant representative meaning for visitors. Ramkissoon et al. classified place attachment into place dependency, affect, identification, and place attachment, indicating that the four aspects of place attachment significantly positively influenced place satisfaction and environmental performance [79]. The intention of the sense of place study was to examine the importance and significance of the tourist destination’s amenities. Tang et al. illustrated that being tied to a place has a positive influence on resource protection and heritage preservation [28]. Using the example of the Chinese Suzhou Garden of WHS, Su et al. demonstrated that the place attachment has a significantly positive influence on the attitudes and behaviors of tourists towards to heritage protection [22]. Williams considered that people’s psychological affection to environment will inspire them to engage in additional responsible environmental behaviors; for example, taking the initiative, picking up garbage, and respecting animals [77]. Meanwhile, related scholars have studied the influencing factors and mechanisms of destination attachment, including tourist crowding and overtourism, destination attractiveness, positive and negative tourist attitudes, and tourist satisfaction and loyalty [39,41,49,55].

More attention has been paid to the social influences of visitors’ place attachment to World Heritage sites as well-known tourist destinations [80]. Relevant research has shown that attachment to tourist destinations has been an important precursor variable of tourism environmental protection and heritage protection intentions, which can change tourist behavior intentions and satisfaction and have positive impacts on heritage protection, while, if there is crowding and other influences, it may cause negative effects [27,55,56,81]. Some scholars have also focused on the destination attachment cycle and the formation procedure of visitors’ attachment to destinations [82,83]. Destination attachment had a positive influence on tourist satisfaction, and cognitive, affective, and conative loyalty [84]. The local connection was an important basis for tourism’s environmental protection intentions and had a significantly positive effect on tourism’s environmentally friendly behavior and the appreciation of travel destinations. It also had a strong correlation with increased destination attachment and satisfaction of tourists [26,73,81]. Williams et al. adopted a psychometric method to gage the efficacy of place attachment, indicating that tourists’ destination attachment results in better comprehension of a destination’s culture and heritage value, which could lead to optimistic psychological consequences and further heritage protection [40]. Tourists’ destination attachment to WHS also plays a key position in tourists’ satisfaction. Relevant research findings imply that destination image and destination attachment have an important positive impact on destination satisfaction, and may enhance tourists’ revisit rate and motivate tourists to better understand and protect OUV and to inherit the culture and heritage values of WHS sites, which are important for WHS conservation and tourist sustainable development in WHS [7,71,85]. Therefore, we propose the hypothesis:

**Hypotheses** **6** **(H6).** *Destination attachment has a positive impact on tourist satisfaction.*

### 2.4. Tourist Satisfaction

Satisfaction is one of the most important concepts in marketing philosophies and study [86]. Satisfaction is a person’s expressive response to a product or service and is the combined result of inconsistent expectations before purchase and perceived performance after purchase. The marketing literature reveals two different viewpoints of the conception of satisfaction: the process and the outcome lookout [87]. Based on the traditional expectancy–disconfirmation theory [88], customer satisfaction is a means to compare what the client hoped and what he/she obtained in a consumption feeling. Satisfaction is a psychological state; if the actual feeling meets or exceeds expectations, customers will feel satisfied, and vice versa [89]. Oliver argues that satisfaction is directly associated to the customer’s perceived satisfaction response and is a judgment about the extent of one’s satisfaction with the service experience [90]. According to the concept of customer satisfaction, visitor satisfaction is described as the level of tourist fulfilment by comparing tourist experience with initial expectations. Satisfaction is probably the most common embodiment of quality-assessment of a recreation experience, and the notion of satisfaction incorporates needs theory, cognitive dissonance theory, and marginal utility theory. In the 1970s, Pizam’s research on satisfaction in tourist destinations laid the foundation. At the end of the 20th century, some scholars engaged in destination marketing, outdoor recreation, and natural and cultural heritage, gradually focus on the application of customer satisfaction theory in tourism, driven by service quality management and customer satisfaction theory. In the 21st century, the concept of tourist satisfaction in tourist destinations has received more attention, reflecting the new concept and trend of “tourist-cantered” tourist management [91]. In the tourism context, individuals leave their usual place of residence in the hope that they will obtain certain desired outcomes at the destination, such as relief of physical and mental fatigue, and understanding of a foreign culture. If the individual finds that perceived performance is greater than the original expectation through tourism experience, then the individual will show a positive emotional response and feel satisfied with the destination. Mechanistically, tourist satisfaction is the same as the customer satisfaction phenomenon, which is a consequence of the association of visitors’ hopes and perceptions; a kind of psychological comparison process results. This also describes the tourist’s psychological occurrence according to the requirement side, which corresponds to the tourism service quality based on the supply side. There are many definitions of visitor satisfaction, indicating that people have different perspectives and depths of understanding. Pizam et al. first found that tourists’ satisfaction is the result of comparing visitors’ expectation and experience at the destination; if the result of comparing experience and expectation makes tourists feel satisfied, then tourists are satisfied; otherwise, tourists are dissatisfied [91]. This theoretical model, which defines visitor satisfaction as the consistency between visitor expectations and actual experiences compared to each other, is widely accepted by the tourism community. Beard et al. further emphasize that visitor satisfaction is a “positive” perception or feeling, based on the positive effect of visitor expectations compared to the actual experience [92]. Based on the Sirgy value reconciliation theory of consumer behavior, it is believed that tourist satisfaction includes functional reconciliation and image reconciliation. Functional reconciliation refers to the reconciliation between tourists’ expectations and perceptions, while image reconciliation refers to the reconciliation between tourists’ self-perceptions and the destination image [93].

Understanding satisfaction is the basis for assessing the presentation of tourism attractions, tourist products, and services, and most studies evaluating consumer satisfaction have used the perceived overall performance approach and models of expectation/disconfirmation [88,90,92]. Leisure satisfaction is controlled by the dissonance amid the consumer’s preferred leisure experience and the actual leisure experience [84]. Tourist satisfaction can be measured by asking them to compare their current tourist destination with other similar sites they have already toured [94]. The tourists’ own assessment of their satisfaction with the travel experience must be taken into account, regardless of their expectations. This means that the actual visitor experience is assessed as post-trip satisfaction, which influences destination choice, product consumption, and services during the trip, as well as decision to revisit [95]. The core elements of satisfaction formation include relationships, give–reward expectations, and perceptions of fairness. Satisfaction plays an important role in predicting behavioral intentions and has a significant role in tourists’ willingness to revisit, community involvement, environmentally responsible behavior, WHS preservation, and support for tourism development [96]. Both visitor satisfaction and behavioral intentions are vital for the achievement of a tourist destination and the goal of its marketing plans [97]. In the tourism industry, satisfaction leads to destination recommendations and increases the likelihood of repeat visits [86]. The satisfaction concept can be tackled from a cognitive perspective, conceptualizing consumer satisfaction as a post-consumption evaluation that either meets or falls short of previous expectations. Conversely, satisfaction could be viewed as the emotional response evoked by consumption, which is the psychological consequence of the visitor after the destination experience [98].

Tourism perceived psychological capacity studies, which have been conducted with the rise of ecotourism and heritage tourism, provide an environmental carrying capacity perspective for the definition of tourist satisfaction. The underlying assumption is that tourist satisfaction decreases as the number of tourists rises, but this approach has been challenged by the unpredictability of perceived carrying capacity. In addition, destination attractiveness is an individual’s cognitive assessment of the performance of a destination, and when the evaluation results in a perceived performance that exceeds the original expectation, the individual becomes satisfied with destination attraction according to the “expectation-inconsistency” framework; perceived value has a significant effect on tourist satisfaction and attitudinal behavior. The authenticity of a WHS affects visitor satisfaction, loyalty, attractiveness, and the assessment of heritage value, and the impact of the protected area’s tourism and service landscape on visitor satisfaction is key to sustainability [99]. It can be said that, e.g., destination perception, relationship quality, environmental behavior are closely related to the outcome of leisure tourism, especially with respect to social satisfaction, physical satisfaction, and overall life satisfaction [100,101]. Related scholars have also studied the combined influences of tourist satisfaction and dissatisfaction, such as the size of the tour and the familiarity of the group, the different types of services, the effectiveness of the interpretation system, the quality of the tour experience, and the crowdedness of the destination [102,103]. Neal et al. analyze visitor satisfaction from a multidimensional and systematic perspective, covering before, during. and after the visit, including destination service satisfaction, travel commuting satisfaction, total effect satisfaction, and total cost satisfaction [104]. Chen et al. examine the combined impact and perceptions of visitors to heritage sites in terms of their quality of experience, perceived value, satisfaction, and behavioral intentions [105]. Defining visitor satisfaction at the holistic level as a cumulative psychological state received from the tourist consumption experience, this conceptualization of visitor satisfaction has been extensively adopted to study the relationship amid satisfaction and other constructs. The quality of the tourism experience amounts to a tourist’s assessment of the degree of merit of his or her tourism experience and a prior variable of the tourist’s satisfaction [102,103]. These visitor satisfaction studies and findings have significant theoretical and practical implications for the experience and interpretation to the OUV and sustainable development of heritage sites.

### 2.5. Conceptual Framework

**Figure 1:**
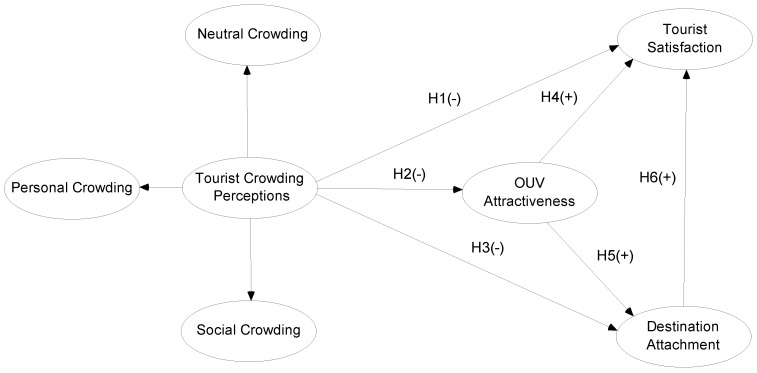
The suggested conceptual framework.

The suggested conceptual framework is shown in Figure 1.

## 3. Methodology

### 3.1. Study Area

**Figure 2:**
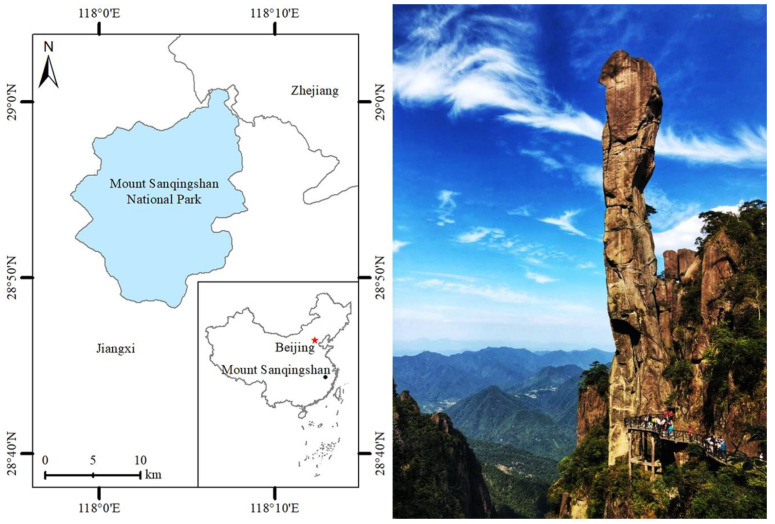
The geographical location and panoramic view of Mount Sanqingshan National Park.

Mount Sanqingshan National Park is situated in Jiangxi Province, PRC, which is named after a traditional Chinese Taoist sacred shrine in Figure 2. Mount Sanqingshan National Park was granted the appellations of World Natural Heritage Site by UNESCO in 2008, national grade 5A tourist attractions (the national top-level tourist destination) in 2011, and World Geopark in 2012, making it a famous tourist destination and WHS in China with several different laurels. The UNESCO’s World Heritage Committee remarks that Mount Sanqingshan National Park features a distinctive set of forested, fantastically shaped granite pillars and peaks concentrated in a fairly small region. Its OUV is exceptional, and it is also a well-known Taoist cultural site with more than 1600 years of history. Since 2000, the tourism industry has developed rapidly, attracting a large number of tourists from home and abroad, and generating favorable economic benefits. The variety of tourists was 580,000 and the tourism revenue was 213 million RMB in 2002, but they soared to 23.95 million and 22.22 billion RMB, respectively, by 2019 in Mount Sanqingshan National Park (http://www.zgsr.gov.cn/sqs/bindex.shtml, accessed on 9 September 2019). Meanwhile, it exerts tremendous pressure on heritage conservation and tourist overload and, inevitably, faces crowding problems and overtourism, which have certain impacts on the perceived attractiveness of OUV and tourist satisfaction, etc., and pose particular challenges to the sustainable development of WHS. Therefore, the selection Mount Sanqingshan National Park as a case site is representative and appropriate.

### 3.2. Measurement Instruments

The scale design is based on the research team’s four comprehensive field surveys and investigations at the case sites, combined with a variety of literature perspectives to develop a synthesis of perception. After pre-survey and ongoing revision, we formed a final formal research questionnaire (Table 1). The questionnaire contained two sections: the first section was divided into demographic characteristics and related tourism organizations, including travel type, number of trips, and length of tourist stay. The second part is a conceptual framework including the four dimensions of tourist crowding perception—perceived OUV attractiveness, destination attachment, tourist satisfaction—and their 28 items. The tourist crowding perception dimension (12 items) was mainly from the related literature and field research, including neutral crowding, social crowding, and personal crowding [41,44,52]. The dimension of OUV attractiveness (6 items) was obtained mainly from related literature [18,106] and WHS selection criteria, as well as the actual expression of the OUV perceptions of heritage site; for example, the scenic spots of ‘a python were coming out the mountain’ is a good representation of the OUV impressions of tourists; hence, we paid more attention to an OUV assessment that aggregated the global context with the local background. The destination attachment dimension (5 items) was mainly received from the related literature [22,40,79,107]. The tourist satisfaction dimension (5 items) was mainly received from the related literature, including willingness to revisit and recommend, and comparison of expectations and actual feelings [52,88,90,92,104]. Scoring for every declaration was accorded to a Likert scale ranging from (1) totally disagree/dissatisfaction to (5) intensely agree/satisfaction.

### 3.3. Sampling and Data Collection

Led by two tutors, the research team consisted of 13 college, postgraduate, and doctoral students who contributed to survey development and/or the poll distribution exercise, and the target group was domestic tourists. From June 9 to June 15 (the period is 7 days a week, visitor satisfaction may be affected due to the different congestion per day), 600 questionnaires were delivered at the chief outlets of Jinsha ropeway and Waishuangxi ropeway in Mount Sanqingshan National Park, of which 591 were recycled and the recovery rate was 98.5%. In order to improve the questionnaire recovery rate and survey quality, we adopted a method in which the two main export outlets were set up in this scenic spot, presented souvenirs, and selected tourists after traveling to Mount Sanqingshan National Park to conduct research. According to the convenient sampling method, the non-serious, incomplete, and illogical questionnaires were deleted, and 536 useable questionnaires were obtained with an effective rate of 90.7%. Meanwhile, we interviewed visitors, scenic resort managers, tourist practitioners, and communities on question such as the tourist interpretation system, OUV protection, and visitor satisfaction to obtain a comprehensive comprehending of the relevant conditions in WHS site.

### 3.4. Data Analysis

This research employed confirmatory factor analysis through structural equation modelling (SEM). SEM permits us to establish, estimate, and test the causality model, and is extensively utilized in social science study. The study performed an investigation of the measurement model, assessed its validity and reliability, and used SEM to identify relationships between the latent constructs. SPSS 22.0 statistical software was adopted to check the collected database. The confirmatory factor analysis and SEM methods were undertaken by means of AMOS 19.0 (SPSS Inc., Chicago, IL, USA). SEM was used to examine whether the framework parameters have an inverse assessments hypothesis, checking the conceptual model to observe whether there was common method bias.

## 4. Results

### 4.1. Sample Profile

As shown in sample characteristics, of the 536 visitors, 42.2% were male and 57.8% were female, approximately 84% were under 40 years old, about 76% had a junior college degree or higher, 59.2% reported earning a monthly salary of 1501 to 5000 RMB, about 85% of the tourists traveled once, 49% stayed one day in the tourist destination, and 42.7% of tourists are accompanied by family and friends and 29.5% by tour group (Table 2).

### 4.2. Reliability and Validity Analysis

Reliability testing is designed to check the reliability, stability, and consistency of scale data. Higher reliability indicates a slighter standard error of measurement. SPSS 22.0 demonstrated that the general robustness of the Cronbach’s alpha scale was 0.90 and the confidence coefficient of the Cronbach’s alpha of every dimension was above the threshold of 0.7. The comprehensive reliability of every dimension is greater than 0.8, signifying that the model is reliable [108]. The sake of the validity testing is to assess the validity of measures scale. The Kaiser–Meyer–Olkin value of the total sample was over 0.91, which indicated reliable construct validity of the questionnaire. The Kaiser–Meyer–Olkin of content validity was greater than 0.70, suggesting that the developed measurement clauses could designate the substance to be measured. An extracted average variance greater than 0.50 signifies that the observed variables could gage the latent variables, and that the convergent validity of every structure is suitable [109]. Additionally, the value of the variance inflation factor (less than 10) demonstrated the absence of multicollinearity in this research [110]. The results indicated that the association coefficients were smaller than the square root of average variance extracted (Table 3), demonstrating that the discriminant validity was reasonable [109].

In conclusion, all indicators are reasonable, and the data gathered was satisfactory (Table 4).

### 4.3. Measurement Model Testing

The structural model was tested and analyzed to investigate the correlation between the variables in the measurement model. At the first, the multivariate normal distribution of the sample was examined. The absolute value of the noticed variable of skewness (0.097–1.264) was smaller than the doorsill of 2.58; the absolute value of the kurtosis (0.289–2.604) was smaller than the doorsill of 10, so the sample data could be considered as a multivariate normality distribution. In addition, the common method bias was examined. Exploratory factor analysis was presented adopting the Harman’s single factor test [111]. The first factor clarified 14.91% of the overall variance, signifying that common method bias was not a serious problem and can be ignored. Thirdly, while checking the total model fit indicators, Hair et al. advocated checking whether there is a violation of the model parameter estimation, which can be ascertained in two ways, i.e., whether there is negative variance of the error or the standardized parameter coefficient is weightier than or equal to 1 [110]. The error variance ranged from 0.014 to 0.072, with no negative error variance in the model. The standardized parameter coefficient ranged from 0.552 to 0.900; it is smaller than 1, signifying that no violation of estimation was ascertained by the goodness-of-fit of the model. Ultimately, we adopted the maximum likelihood technique to evaluate the parameters of the theoretical model. We found that the related fitting parameters are not perfect, and needed to make further modifications. In accordance with the principle of releasing one at a time, NC1 was associated with NC2, OA1 with OA2, PA1 with PA2, and TS4 with TS5 according to the modified change values. The modified structural model-fit indices were comparatively suitable (Table 5) [79].

### 4.4. Structural Model Testing

**Figure 3:**
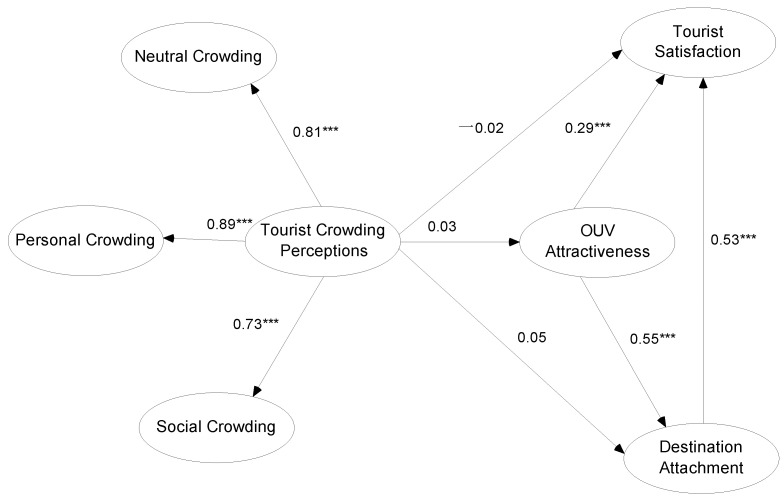
Results of the structural model analysis. Note: *** Significance at 0.001 level.

The hypothetical causal correlations were checked. The outcomes of the assessment are presented in Figure 3 and Table 6.

## 5. Discussion

### 5.1. Discussion of the Results

The outcomes of the research hypotheses were show in Figure 3 and Table 6, where the hypotheses of H1, H2, and H3 were rejected, and the hypotheses of H4, H5, and H6 were supported, respectively.

In this study, H1, H2, and H3 were rejected; the influences of tourist crowding perception on OUV attractiveness, destination attachment, and tourist satisfaction were not remarkable, indicating that the degree of perceived crowding generated by tourists visiting Mount Sanqingshan National Park is not high, and had no negative impact on visitor satisfaction. This may have something to do with the fact that the heritage site was not very heavily visited during the research period and was not very overcrowded itself. After more than 20 years of tourist development in Mount Sanqingshan National Park, especially since it was listed as a WHS, the quality of supporting services related to the destination has been greatly improved, and the level of protection and management of heritage sites and tourist governance has been greatly enhanced. In the meantime, Mount Sanqingshan National Park has also made special plans for visitor management and smart tourism development, which has improved visitor satisfaction and loyalty to a certain extent. This is also consistent with the findings that crowding does not necessarily have a negative impact, but to some extent diminishes visitor satisfaction [52,56,58,59]. General research, however, concludes that crowding inevitably has a negative influence on tourist satisfaction; overcrowding and overtourism in particular become major reasons for tourist satisfaction and loyalty, and overcrowding has a substantial negative impact on tourist destination satisfaction and willingness to visit again [10,15,52,57]. Meanwhile, research findings suggest that moderate crowding may have a positive impact and that crowding is not always a terrible outcome [49,50]. The association between visitor crowding perceptions and satisfaction also has a connection with the quality of visitor experience and perceived environment such that the degree of negative physical environment characteristics is closely related to tourist satisfaction [9]. In this study, Chinese tourists are used as a case study, and since China has a large population and is relatively amenable to crowding, moderate crowding may not have an important effect on visitors; the mean value of the tourist crowding perception dimension is 3.38 and is characterized by a five-level Likert gage advocated by Tosun (the mean values ranged 1.0 to 2.4 are objection; 2.5 to 3.4 are neutral; 3.5 to 5.0 are agreed), indicating a neutral attitude to crowding perception [112]. In addition, the mean value of the social crowding dimension is 3.24, indicating that Chinese people are more socially inclusive in their crowding perception. Thus, the hypothesized role of crowding on OUV attractiveness and destination attachment for H2 and H3 is not significant, perhaps for the same reason. There are relevant results that crowding perceptions and overtourism have a negative impact on destination attractiveness [15,17], yet also have a positive emotional impact [50]. We speculate whether it is also associated with the OUV of Mount Sanqingshan National Park; with a glimpse of the stunning beauty of the WHS, the negative perception of tourist crowds, etc., may, in this moment, cease to exist, which is perhaps the charm of OUV.

The results of hypothesis H4 show that the OUV attractiveness of heritage sites has a significant positive influence on destination attachment, indicating that the powerful charm and mind-blowing beauty of a WHS have a significant impact on tourists, fully validating the core attractiveness of the OUV (mean value is 4.05, signifying that visitors’ perception of OUV is amazing and approved); just as the ancient poem says there is no need to go looking for any fairy dwelling, this is a fairyland on earth (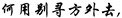

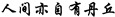
, There is no need to look for other places to go, where is also a Gods living on earth.), which corelates with the results of related research [39]. At the same time, OUV attractiveness helps to improve visitor satisfaction, enhance visitor loyalty, and increase the revisit rate, as concluded in hypothesis H5, indicating that OUV attractiveness has a significant positive influence on visitor satisfaction. This is consistent with the results of related research, which show that the charm of OUV and the beauty of heritage sites have a significant effect on visitor experience, visitor emotion, and visitor satisfaction, which also contributes to heritage conservation and the interpretation and dissemination of OUV and is important for the tourism sustainable development [64,66]. Tourism experience is the outcome of a combination of factors, including the attractiveness of the destination, service quality, management level, etc. Although destination attachment is formed under a brief tourism experience, it still has an important human–place relationship, and a sound human–place relationship is conducive to enhancing tourists’ environmental awareness and heritage conservation behavior [13,71,81,83]; just as in the results of hypothesis H6, destination attachment has a significant positive influence on visitor satisfaction and the mean value of the destination attachment dimension is higher (4.21), which fully indicates that tourists have a strong destination attachment to WHS. Although tourists and their journey destinations are only a short, off-site human interaction link in a non-habitual environment, it has a certain contribution to tourists’ satisfaction, environmental behavior, and in-depth experience of the destination, which is in line with the relevant research judgements [22,28,39,41,49,55]. In addition, the correlation among destination attachment and visitor satisfaction is reciprocal in both directions, i.e., relevant studies have shown that tourist satisfaction has a significant influence on place attachment [7,71,83]; the mean value of visitor satisfaction was 3.90, indicating a low negative effect of perceived crowding and even no negative influence on OUV attractiveness and destination attachment, proving a high level of visitor satisfaction with WHS trip.

In conclusion, the hypothesis that tourist overcrowding perceptions have a significantly positive influence on tourist satisfaction, OUV attractiveness, and destination attachment were not supported, suggesting that tourist overcrowding perceptions do not necessarily have an adverse effect. In addition, OUV attractiveness has a significant positive influence on tourist satisfaction and destination attachment, and destination attachment significantly impacts tourist satisfaction. OUV attractiveness, destination attachment, and tourist satisfaction together have a significantly positive influence and the quality of overall tourist experience is higher.

### 5.2. Theoretical and Managerial Implications

From a theoretical viewpoint, this study explores the effects of crowding perception on OUV attractiveness, destination attachment, and tourist satisfaction as antecedent variables, and the influence of OUV attractiveness on visitor satisfaction, which provides a new angle for analysis of tourist satisfaction and OUV value perception investigations in WHS. The conclusion of study did not emphasize the negative effects of crowd perception, which differs from most previous studies that have found that crowd perception inevitably has adverse or negative consequences. In particular, the positive impact of OUV attractiveness on destination attachment and satisfaction suggests that OUV is a core value of WHS, that strong charisma is a driving force to visitor satisfaction, and that a short journey generates destination attachment and emotional connection to WHS. Our study emphasizes the importance of OUV interpretation, communication and conservation, not only for the heritage site itself, but also for visitors, who are important stakeholders in heritage tourist destination. This thesis provides another conceptual paradigm within which to explore the attitudinal behavior of visitors to WHS and the cultural-tourism transformation of OUV.

From the view of WHS conservation and heritage tourism management, the quality of tourists’ experience at heritage sites should be paid more attention; the carrying capacity of tourist destination and tourists’ volume, in particular, should be controlled within an appropriate scale, so as to effectively manage the crowdedness of WHS, which is conducive to tourists’ better appreciation of the charm of OUV and enhance the emotional attachment and loyalty to WHS, thus reducing public sentiment and pressure to heritage conservation. Meanwhile, we have noticed that the role of OUV is very prominent, so we should better explore, interpret, elaborate, and promote OUV. On the one hand, we can make tourists perceive the charm of OUV more effectively through tourism interpretation and display systems after they arrive at destination, combined with integrated media technology; on the other hand, we should give tourists sound OUV science education when promoting and publicizing tourism products at heritage sites, and a deeper understanding may better boost heritage site conservation. In particular, the advantages of technologies such as smart tourism, virtual tourism, and artificial intelligence are performed before, during, and after the experience of tourism to give tourists a multi-dimensional perception of OUV, which can be conducted through heritage conservation education and heritage science popularization, improving the quality of tourism experience, promoting the value of heritage tourism, controlling the capacity of heritage sites, and decongesting visitors’ crowding, with a view to contributing to the manifestation of OUV value and sustainable development of heritage sites.

### 5.3. Limitations and Prospect Directions

Relevant findings were obtained; however, there are related limitations and topics to be researched in future study. First, the experience, assessment, and influencing factors of tourists’ satisfaction should be established in a multidimensional measurement model, which can be studied more comprehensively and systematically from the perspectives of tourism service quality, tourism value fulfillment, tourism capacity perception, tourism preference, etc. Secondly, the measurement of tourists’ OUV attractiveness perceptions is best combined with the level of tourists’ heritage education and the efficacy of the interpretation display system, so that the assessment and manifestation of OUV value can be carried out more accurately and reasonably. Third, to acquire an objective and site-specific approach to visitor crowding perceptions, future research can incorporate spatial and temporal contexts, focus on differential measures of visitor crowding, identify key influencing factors, and explore the multifaceted impacts, such as the effects of visitor crowding on heritage site management, destination planning, and sustainable tourism development in WHS. Fourthly, the research on tourists’ destination attachment should pay more attention to the antecedent influencing factors of its role and bring its positive value for heritage conservation and other aspects into play, which can then be afforded a systematic study from the perspective of subject and guest sharing and value co-creation. Fifthly, the quality of tourism experience is not only judged by either/or criteria, just as tourist crowding does not necessarily have negative effects. In fact, humans do not always act in accordance with rational principles and are not only “economic agents”, but perhaps act more in line with the concept of reciprocity theory, which opens the door to co-creation of value and mutual synergy for tourist satisfaction. Ultimately, this investigation is founded on the Chinese cultural context and traditional philosophical ideas, the case land being the Taoist destination. Taoist thought are extremely influential to some extent in China, particularly this doctrine that human beings should respect and follow nature and that the relationship between human and earth should be harmonious (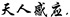

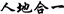
, celestial–human induction, human–earth unity); whether and how much these have an influence on tourist crowding perceptions, OUV attraction, destination attachment, etc., are topics worth exploring in future. In future studies, we should also consider the effects of service quality, level of destination management, and unexpected crisis events (e.g., COVID-19 epidemic) on visitor attitudes and behaviors, which in turn may affect visitors’ willingness and behavior regarding heritage tourism.

## 6. Concluding Remarks

On the basis of OUV attractiveness and tourist crowding of Mount Sanqingshan National Park, a theoretical framework of tourist satisfaction is constructed based on the visitor perception perspective in the WHS site, and destination attachment is incorporated to investigate the influencing factors and mechanisms of tourist satisfaction. First, tourist crowding perception in WHS has no significant negative effect on perceived OUV attractiveness, destination attachment, and tourist satisfaction, and tourist perception of crowding is generally low, which distinguishes this study from most relevant academic studies that conclude that crowding necessarily has an adverse effect on tourist satisfaction. Second, the OUV attractiveness has a significant positive effect on tourists’ destination attachment and satisfaction, fully demonstrating the charm of WHS sites’ OUV, which is also a core resource endowment for tourist development, indicating that OUV performs a pivotal role in tourists’ destination attachment, destination reputation, satisfaction, loyalty, and even revisit rate, and that OUV is a core attraction that deserves its name. Third, visitor satisfaction positively contributes to tourism development in WHS, facilitating heritage conservation and the interpretation and promotion of OUV values. Dialectically, satisfaction in turn enhances tourist destination attachment and willingness to protect OUV, further reducing negative impacts such as tourist congestion, which is also mutually beneficial and synergistic. Fourth, the conceptual framework proposed in this study expands the vision for visitor satisfaction in WHS and has a certain theoretical significance. It is also a positive response to the strategic goal of the 5Cs (credibility, communication, capacity-building, conservation, and community) of WHS, especially for the credibility, communication, and capacity-building of heritage sites, and has a certain practical guidance implication for heritage conservation and tourist management in WHS sites.

## Figures and Tables

**Table 1:** Constructs and measurement items.

Constructs	Items		
Tourist Crowding Perceptions (TCP)	Neutral Crowding (NC)	NC1	Tourist perceived crowding of the trail ways in MSNP
		NC2	Tourist perceived crowding of the resting areas in MSNP
		NC3	Tourist perceived crowding of the tourist toilets in MSNP
		NC4	Tourist perceived crowding of the catering services in MSNP
	Personal Crowding (PC)	PC1	Many visitors interfere with my usage of public facilities
		PC2	Many visitors hindered me from enjoying the scenery
		PC3	Many visitors made me feel crowded with space
		PC4	Many tourists disturbing public order made me upset.
	Social Crowding (SC)	SC1	I was surrounded by too many strangers to spoil the enjoyment of the scenery.
		SC2	I felt nervous due to enclose by numerous visitors
		SC3	I felt uneasy due to contact with numerous visitors
		SC4	I believe the wildness was missing since so many visitors here.
OUV Attractiveness (OA)	OA1	The natural landscape is mesmerizing in MSNP.	
	OA2	The magnificent peaks and rocks are amazing in MSNP.	
	OA3	The rapids and waterfalls are exciting in MSNP	
	OA4	The gorges and clouds are intoxicating in MSNP.	
	OA5	The Geomorphology and landscape is peculiar in MSNP.	
	OA6	The scenic spots of ‘a python were coming out the mountain’ is awesome	
Destination Attachment (DA)	DA1	Tourist order of MSNP is comfortable	
	DA2	Tourist atmosphere of MSNP is friendly	
	DA3	The MSNP is a place to stay in love	
	DA4	The MSNP is a place to stay in memory	
	DA5	The scenery of MSNP is unique.	
Tourist Satisfaction (TS)	TS1	How satisfied are you with the scenic area in general?	
	TS2	How satisfied are you with your expectations before the tour?	
	TS3	How satisfied are you compared to your ideal destination?	
	TS4	Would you like to revisit MSNP?	
	TS5	Would you like to recommend others to visit MSNP?	

Note: Mount Sanqingshan National Park, MSNP.

**Table 2:** Sample characteristics.

Items		%	Items		%
Gender	Male	42.2	Income (RMB /month)	≤1500	17.1
	Female	57.8		1501–3500	29.3
Age	≤20	9.2		3501–5000	29.9
	21–30	49.9		5001–8000	12.8
	31–40	24.9		≥8001	10.8
	41–50	13.4	Travel manner	Family, friends, etc.	42.7
	≥51	2.4		Tour group	29.5
Profession	Enterprise personnel	35.5		Organizational travel	13.8
	Professionals, civil servants	22.8		Travel alone	6.4
	Student	16.2		Others	7.6
	Worker, farmer	9.3	Tour times	Once	85.5
	Others	16.2		Twice	7.1
Education	Middle school or below	7.1		Three times or more	7.4
	High school	17.3	Stay days	One day	49.1
	College graduate	68.3		Two days	35.2
	Postgraduate	7.3		Three days or more	15.7

**Table 3:** The discriminant validity of latent variables.

Variable	TCP	OA	PA	TS
TCP	0.810			
OA	0.023	0.769		
DA	0.074	0.558	0.747	
TS	0.033	0.570	0.698	0.776

Note: TCP, tourist crowding perceptions; OA, OUV attractiveness; DA, destination attachment; TS, tourist satisfaction.

**Table 4:** Means, reliability, and convergent validity.

Variables	Items	Mean	Standard Deviation	Standardised Loading	Cronbach’s Alpha	Average Variance Extracted	Combination Reliability
Tourist Crowding Perceptions	NC	3.489	1.384	0.810	0.929	0.656	0.851
	PC	3.404	1.405	0.869			
	SC	3.239	1.635	0.746			
OUV Attractiveness (OA)	OA1	4.195	0.978	0.802	0.895	0.591	0.896
	OA2	4.141	0.979	0.824			
	OA3	3.653	1.163	0.648			
	OA4	4.079	1.042	0.803			
	OA5	4.188	0.944	0.789			
	OA6	4.073	1.056	0.734			
Destination Attachment (DA)	DA1	4.098	0.885	0.552	0.861	0.558	0.859
	DA2	4.203	0.845	0.622			
	DA3	4.258	0.840	0.900			
	DA4	4.229	0.855	0.887			
	DA5	4.275	0.828	0.707			
Tourist Satisfaction (TS)	TS1	3.869	0.723	0.722	0.882	0.602	0.883
	TS2	3.794	0.830	0.822			
	TS3	3.817	0.829	0.831			
	TS4	3.890	0.853	0.727			
	TS5	4.109	0.764	0.772			

Note: NC, neutral crowding; PC, personal crowding; SC, social crowding.

**Table 5:** Goodness-of-fit indices.

Model-Fit Index	Absolute Index			Comparative Index			Parsimony Index		
	CMIN/DF	GFI	RMSEA	IFI	TLI	CFI	PGFI	PNFI	PCFI
Threshold value	1–3	>0.90	<0.08	>0.90	>0.90	>0.90	>0.50	>0.50	>0.50
Theoretical model	3.148	0.876	0.063	0.928	0.920	0.928	0.735	0.810	0.837
Revised model	1.919	0.920	0.041	0.970	0.966	0.970	0.764	0.837	0.864

**Table 6:** Overview of hypotheses analysis results.

Hypotheses				SRW	C.R.	Outcomes
H1	Tourist satisfaction	<---	Tourist crowding perceptions	−0.021	−0.556	Rejected
H2	OUV attractiveness	<---	Tourist crowding perceptions	0.032	0.631	Rejected
H3	Destination attachment	<---	Tourist crowding perceptions	0.051	1.166	Rejected
H4	Destination attachment	<---	OUV attractiveness	0.555 ***	10.430	Accepted
H5	Tourist satisfaction	<---	OUV attractiveness	0.289 ***	5.863	Accepted
H6	Tourist satisfaction	<---	Destination attachment	0.529 ***	9.566	Accepted

Note. *** Significance at 0.001 level.

## Data Availability

No data available to share at this time.
